# Serological Markers of Viral Infections (Rubella Virus, Human Cytomegalovirus and Arboviruses) among Symptomatic Pregnant Women in Rural and Urban Areas of Mwanza, Tanzania

**DOI:** 10.3390/tropicalmed6040186

**Published:** 2021-10-19

**Authors:** Najma Awadh, Helmut Nyawale, Elieza Chibwe, Fridolin Mujuni, Margareth Ollomi, Karim Hassan, Majigo Mtebe, Lucas Matemba, Stephen E. Mshana, Mariam M. Mirambo

**Affiliations:** 1 Department of Obstetrics and Gynecology, Weill Bugando School of Medicine, Catholic University of Health and Allied Sciences, Mwanza 1464, Tanzania; candy-naj@yahoo.com (N.A.); eliezachibwe@yahoo.com (E.C.); frido2mj@gmail.com (F.M.); 2 Department of Microbiology and Immunology, Weill Bugando School of Medicine, Catholic University of Health and Allied Sciences, Mwanza 1464, Tanzania; helmutny@yahoo.com (H.N.); margarethollomi@gmail.com (M.O.); karimabdulrahman20@gmail.com (K.H.); mshana72@bugando.ac.tz (S.E.M.); 3 Department of Microbiology and Immunology, Muhimbili University of Health and Allied Sciences, Mwanza 65001, Tanzania; mmajigo@gmail.com; 4 National Institute for Medical Research Headquarters, Dar es Salaam 9653, Tanzania; lmatemba@yahoo.com

**Keywords:** rubella virus, human cytomegalovirus, Zika virus, dengue virus, pregnant women, Tanzania

## Abstract

Viral infections have been associated with poor pregnancy outcomes. We investigated the magnitude of rubella virus (RV), dengue virus (DENV), Zika virus (ZIKV) and human cytomegalovirus (HCMV) among symptomatic pregnant women in rural and urban areas of Mwanza. A cross-sectional study was conducted between July 2017 and April 2018 in Mwanza. A rapid immunochromatographic test was done to detect ZIKV IgM and IgG as well as DENV IgM and IgG antibodies. A multiplex_RT-PCR was also done to detect the viral RNA genome. Enzyme immunoassays were done to detect RV and HCMV. Out of 171 participants, 1 (0.6%) was found to be seropositive for ZIKV_IgM antibodies, while 5 (2.9%) were ZIKV_IgG seropositive. DENV seropositivity was 9 (5.3%) and 3 (1.8%) for IgM and IgG, respectively, with all being PCR negative. Two participants (1.2%) were RV_IgM seropositive. 100% were HCMV_IgG seropositive and none was HCMV_IgM seropositive. Among 70 women with high HCMV_IgG titters, 10 (14.3%) had a low avidity index, indicating recent infections. Residing in rural areas (*p* = 0.044) and advanced age (*p* = 0.024) independently predicted ZIKV/DENV seropositivity. A substantial proportion of pregnant women had markers for viral infections. There is a need for introducing routine screening and monitoring pregnancy outcomes of positive cases to establish the relationship of these viruses and adverse pregnancy outcomes in endemic areas.

## 1. Introduction

Viral infections during pregnancy have been considered benign, with a few notable exceptions, such as Human Immunodeficiency virus (HIV). Nevertheless, recent findings and outbreaks have revealed that pregnant women often suffer adverse outcomes, ranging from spontaneous abortions, preterm labour, stillbirths and various forms of congenital malformations [1,2,3]. Interactions between the fetal–maternal interface and the immune system play a key role in these outcomes as a result of the immunological changes taking place during pregnancy.

In this era, when we are confronting great risks for pandemics, understanding paradigms of these infections during the perinatal period is of great importance. Overwhelming evidence suggests that perinatal viral infections are common in resource-constrained countries and significantly contribute to the adverse pregnancy outcomes [4,5,6,7,8]. Rubella virus (RV), human cytomegalovirus (HCMV), dengue virus (DENV) and Zika virus (ZIKV) are endemic in the tropical regions and have been fairly studied in different populations in the study setting, with IgG seroprevalence of RV and HCMV of more than 90% being reported among general pregnant women [9,10,11,12,13,14]. Recently, in a study involving 8 districts, the seroprevalence of CHIKV, DENV and ZIKV were 28%, 16.1% and 6.8%, respectively. The seroprevalences of CHIKV, DENV and ZIKV in the Ukerewe district, Mwanza were 43.4%, 12.8% and 10.6%, respectively [15]. Despite being endemic in these countries little has been documented regarding the magnitude and outcomes associated with these infections during pregnancy. Notwithstanding, a wide range of clinical manifestations that are associated with these viruses, with the majority sharing signs and symptoms and with some cases presenting without any notable symptoms. The most common symptoms during pregnancy include fever, rash, joint pains and myalgia, among many others [5,16,17].

RV infection has been causing congenital rubella syndrome (CRS) [5,18], with about 100,000 children born with CRS each year, especially in resource-constrained countries [19]. Due to this, the government of Tanzania introduced two doses of measles/rubella vaccination at 9 months and 18 months of age in 2015. Similarly, HCMV primary infections pose a great risk to a developing fetus and newborns [20], with about 20–25% risk of adverse pregnancy outcomes [21,22], including 10% chance of fetal deaths [23,24]. Furthermore, DENV and ZIKV have also been associated with adverse pregnancy outcomes [1,25]. In a recent ZIKV outbreak in Brazil, increased cases of microencephaly and other neurological disorders was noted and linked to ZIKV infections [26]. A recent systematic review reported several cases of antenatal complications associated with DENV, including miscarriage, stillbirth and premature deliveries [2]. In addition, maternal death due to DENV infection in pregnancy has been reported in Brazil [27,28].

Despite their importance, the magnitude of these infections during pregnancy remains poorly understood in many low- and middle-income countries (LMICs), including Tanzania. Most available data have focused on general populations and normal pregnant women. Few reports on RV and HCMV exist in Tanzania, without a single report on ZIKV and DENV viruses in pregnant women. The current study focused on establishing sero-epidemiological information regarding these viruses among pregnant women reported to have any of the common symptoms of these infections in the current pregnancy at the time of commencement of this study. Early detection and fetal monitoring of these infections during pregnancy are important options to be considered in the antenatal packages, especially in endemic regions.

## 2. Methodology

### 2.1. Study Design, Study Area and Duration

Between July 2017 and April 2018, a cross-sectional hospital-based study was conducted in rural and urban antenatal clinics in the Mwanza region. The study was conducted in the Sengerema, Nyamagana and Ilemela districts. Mwanza is located in the North-West zone of Tanzania, with a population of 2,772,509 people [29]. This study was conducted at Sengerema Designated District hospital (SDDH), which has a bed capacity of 320 and attends approximately 30–40 pregnant women daily. Another place was Makongoro Health Centre, a primarily reproductive and child health care facility located in the Nyamagana district that has a population of 363,452. This clinic serves both districts, i.e., Nyamagana and Ilemela, with an estimated attendance of 60–100 pregnant women in each day. Furthermore, in the Ilemela district with a population of 343,001 people [30], the study was conducted at Karume Health Centre, which is 20 km from the city of Mwanza and serves about 40–60 pregnant women daily.

### 2.2. Study Population and Selection Criteria

All pregnant women, at different ages and gestation ages, presenting/reported to have at least one of the signs or symptoms consistent with RV, HCMV, ZIKV and DENV infection (fever, rash myalgia/joint paints), in the current pregnancy, attending selected reproductive and child health (RCH) clinics in Mwanza, were enrolled in the study. All women with established causes of these symptoms, such as malaria, were excluded.

### 2.3. Sample Size Estimation and Enrollment

Sample size was calculated using the Kish Leslie formula; a dengue prevalence of 12.5% [31] was used. The minimum sample size was 168; however, a total of 171 women were enrolled.

### 2.4. Data Collection, Sample Collection and Laboratory Procedures

Participants were interviewed to collect sociodemographic characteristics, relevant medical history and associated factors using semi-structured questionnaires. About 5 mL of blood was collected aseptically using a needle and syringe, and it was kept in plain sterile vacutainer tubes (Hanchuan Fumo Plastics Co., Ltd., Jiangsu, China). Samples were transported to the CUHAS microbiology laboratory, and sera were extracted from whole blood by centrifuging at 3000 rpm for 5 min. Sera were stored in cryovials at −80 °C until processing.

All samples were tested for ZIKV and DENV using a multiplex reverse transcriptase quantitative polymerase chain reaction (RT-qPCR), as previously described [32]. Detection of ZIKV and DENV IgM- and IgG-specific antibodies was done using a rapid immunochromatographic test, as per the manufacturer’s instructions (Bio-Scan Medical Laboratories Ltd., Vancouver, Canada). The assay has a sensitivity of >90.0%, and a specificity of 99% and has been recommended for screening ZIKV in Canada (https://cdn.who.int/media/docs/default-source/blue-print/zika-dx-landscape-report.pdf, accessed on 22 September 2021). These assays test for ZIKV at the same time that they test for DENV, as these viruses tend to cross react, hence ruling out cross reactivity. RV IgM antibodies were detected by a mu capture enzyme linked immunosorbent assay (PISHTAZ TEB DIAGNOSTICS, Iran), while HCMV antibodies were detected using an indirect ELISA (Qingdao Hightop Biotech Co. Ltd., Qingdao, China). These ELISA assays have a specificity and sensitivity of >99%. The HCMV IgG avidity assay was performed as described previously [33], using the 6M urea as washing buffer.

### 2.5. Study Variables and Data Analysis

The dependent variables were the presence of RV-, HCMV-, ZIKV- and DENV-specific antibodies, while the independent variables were sociodemographic and other relevant clinical information (such as age, gestation age, education level, residence, signs and symptoms, etc.). Data were entered into a computer using Microsoft Office Excel 2016, and descriptive analysis done using STATA version 13. Categorical variables were summarized as proportions and analyzed using the Pearson’s Chi-square test to observe the significant differences in the distribution of outcomes in various groups. Continuous variables were summarized as a median with an inter-quartile range. Univariate and multivariate logistic regression was done to determine the associated factors for ZIKV/DENV seropositivity. Variables with *p*-value of less than 0.2 on univariable regression analysis and those with biological plausibility were inputted into the multivariable logistic regression analysis. The odds ratio and 95% confidence interval were noted. Predictors with a *p*-value of less than 0.05 were considered statistically significant.

## 3. Results

**Figure 1:**
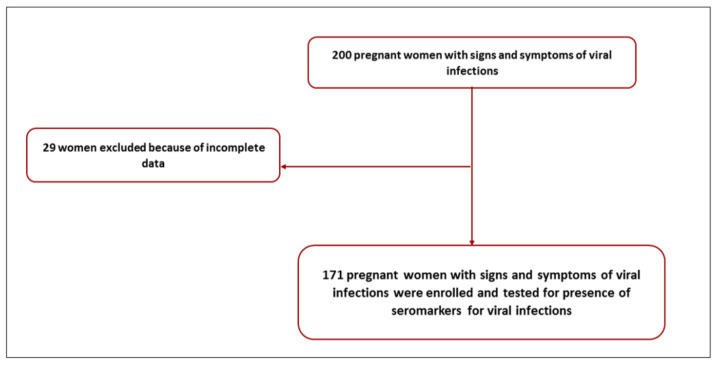
Enrollment flow chart.

During the study period, a total of 200 women with signs and symptoms of RV, HCMV, ZIKV and DENV infections in the current pregnancy were enrolled. Twenty-nine (Figure 1) women were excluded because they were not sure of the gestation age of the current pregnancy, and only 171 were enrolled and tested for the presence of the markers indicating exposure to these viruses. It should be noted that by the time of enrollment, the majority (77.8%) of these women had no signs and symptoms.

### 3.1. Sociodemographic Characteristics of the Enrolled Pregnant Women (N = 171)

The median age of enrolled women was 24 [IQR: 21–30] years, and the median gestation age was 25 [IQR 20–32] weeks. The majority (154, 90.1%) of women were married, and 139 (81.3%) had attained primary education. According to the city layout, 116 (32.2%) were from rural areas, whereas 55 (67.8%) were from urban areas. Of the 171 participants, 81 (47.8%) were small-scale business women (Table 1).

### 3.2. Clinical Features of Viral Infections among Symptomatic Pregnant Women (N = 171)

The most common clinical features were myalgia (158, 92.4%), joint pain (149, 87.1%) and fever (145, 84.8%) (Table 2).

### 3.3. Prevalence of Markers for Viral Infections among Pregnant Women (N = 171)

Out of 171 participants, only one (0.6%) was found to be ZIKV IgM seropositive, while 5/171 (2.9%) were ZIKV IgG seropositive. Regarding DENV, 9/171 (5.3%) were IgM seropositive, and 3 (1.8%) were IgG seropositive, making the overall seroprevalence for DENV to be 12 (7.0%). The seroprevalence of either ZIKV or DENV was 16/171 (9.4%, 95% CI: 5.0–13.7). Only one sample was IgM DENV positive and IgG ZIKV positive. None of the tested samples was found to be ZIKV/DENV positive by the multiplex polymerase chain reaction (PCR). Out of 171, only 2 (1.2%, 95% CI: 0.4–2.8) tested seropositive for RV IgM antibodies. Regarding HCMV, all women (171, 100%) tested positive for HCMV IgG antibodies, while none of them was HCMV IgM seropositive. Out of 171 HCMV IgG seropositive women, 70 were found to have high IgG titers. Among 70 women with IgG titers, 10 (14.29%) were found to have a low avidity index, indicating recent infections (Table 3).

A total of 133 (77.8%) women enrolled had a history of symptoms in the current pregnancy but not at the time of enrollment. Out of those with signs and symptoms at the time of enrollment, only one was DENV IgM seropositive.

### 3.4. Factors Associated with ZIKV/DENV among Symptomatic Pregnant Women in Mwanza

On the Wilcoxon (Mann–Whitney) ranksum test, there was no significant difference between the median age of ZIKV/DENV seropositive women and ZIKV/DENV seronegative women (29 [IQR: 24–34.5] vs. 24 [21–30], *p* = 0.887). Furthermore, no significant difference was observed between the median gestation age (GA) of ZIKV/DENV seropositive (28, IQR: 21–35) and ZIKV/DENV seronegative (25, IQR: 20–32) women (*p* = 478). In addition, out of 116 women from rural areas, 14 (12%) were ZIKV/DENV seropositive, compared to 2 (3.6%) of 55 women from urban areas (*p* = 0.096). By multivariable logistic regression analysis, increase in age (OR = 1.16, CI: 1.02–1.32, *p* = 0.024) and residing in rural areas (OR = 5.03, CI: 1.04–25.29, *p* = 0.044), independently predicted ZIKV/DENV seropositivity (Table 4).

### 3.5. Outcome of Symptomatic Pregnant Women (N = 171) Attending Antenatal Clinics in Mwanza

The majority (152, 88.9%) of participants had normal pregnancy outcomes, including those who were ZIKV seropositive. Among the adverse outcomes, prenatal death (7, 4.1%) was the leading outcome, followed by still-birth (4, 2.3%) and abortion (3, 1.8%). Three out of four women with stillbirths had low avidity HCMV IgG, and among women with abortions, one was Dengue virus IgM seropositive, and two had low avidity HCMV IgG.

## 4. Discussion

This has been the first study to investigate RV, HCMV, ZIKV and DENV seropositivity among symptomatic pregnant women in Mwanza, Tanzania. In this study, seropositivity of specific ZIKV IgG and IgM antibodies were found to be 2.92% and 0.6%, respectively, while none of them was positive for ZIKV and DENV by PCR. This observation is comparable to the study done in Brazil that showed ZIKV IgG seropositivity of 0.55% among blood donors with negative PCR results [34]. The seropositivity of ZIKV IgG in the current study was slightly higher than that of Brazil among blood donors; the possible explanation could be differences in the study population, whereby the current study focused on pregnant women with signs and symptoms consistent with ZIKV infection. Negative PCR results in this study could be explained by the fact that, in the current study, most of enrolled women reported a history of rash during the current pregnancy and not at the time of enrollment. Previous studies [35,36,37] reported that samples collected around the time of rash have a high positive predictive value compared to samples collected at other times of illness. ZIKV and DENV have a short duration of viremia; hence, in most cases, the PCR assays are likely to be negative if samples are collected more than 7 days after the onset of rash [35,38]. Future studies should ensure that samples are collected around the rash period. In comparison with previous studies in Brazil and French Polynesia that reported the prevalence of 53% and 73%, respectively [39,40], the reported seropositivity in this study is indeed low. This is due to the fact that the previous studies were carried out during the ZIKV outbreak.

The reported seropositivity of IgG and IgM for DENV in the current study was significantly lower than 44% reported in Brazil [41]. The difference could be due to the fact that the study in Brazil used notification forms of suspected Arbovirus infection cases during pregnancy from the Brazilian Notifiable Information System for Notification of Diseases (SINAN), which was not the case in the current study.

Regarding RV, 1.2% of studied women had an acute infection, which is comparable to a previous study in the same settings that reported an IgM seropositivity of 0.3% among asymptomatic pregnant women [42]. This unexpected low seropositivity in the current study could be explained by the fact that signs and symptoms of acute RV infections are nonspecific, and most of the time, they mimic those of other viruses, which have been reported to be common in Mwanza [6,43,44]. In addition, the majority of the women enrolled in this study were reported to have signs and symptoms whereby some of them might have occurred a long time before sample collection. Further studies should focus on women with signs and symptoms during enrollment to the study. In comparison with a previous study by Lulandala et al., among women with spontaneous abortion in the same setting, which reported the prevalence of 3.7%, the reported seropositivity in the current study is low [45]. The possible explanation could be the differences in the study population, whereby the previous study focused on women with spontaneous abortions. Results from the current study are also comparable to other African countries, such as Northwestern Nigeria and Ethiopia, which reported a seropositivity of 2.6% and 21%, respectively [46,47]. These findings are also similar to those reported in Asian countries, such as Bangladesh and Turkey, which reported a seropositivity of 2% and 0.75%, respectively [48,49].

The seropositivity of specific HCMV IgG antibodies in this study was found to be 100%. These findings are in agreement with a previous study in Nigeria among pregnant women attending antenatal clinics [50]. This seropositivity is indeed higher than the data previously reported in France [51] and Durango city [52], which reported a seropositivity of 46.8% and 65.6%, respectively. In comparison with a previous study in the same setting among normal pregnant women, the reported seropositivity in the current study is significantly high [6]. The high seropositivity of HCMV IgG antibodies among pregnant women with signs and symptoms in the present study may be explained by the population studied, whereby women who reported to have signs and symptoms in the current pregnancy were enrolled. Regarding IgM seropositivity, in the current study, none of the participants was IgM seropositive, which is different from a study conducted in the same setting [6] and West Sudan [53], which reported an IgM seropositivity of 0.4% and 3.4%, respectively. In this study, none of the factors was associated with HCMV seropositivity, which is similar to a previous study among pregnant women in Nigeria [53]. In the current study, more than one tenth of women were found to have recent HCMV infection, based on the avidity assay. Vertical transmission has been documented in patients who are IgM seronegative with a low IgG avidity index in a previous study [54]. HCMV IgG avidity testing is a reliable tool for detecting primary HCMV infection during pregnancy. Low avidity indicates primary infection, with an increased risk of vertical transmission to the fetus. High avidity in the first trimester excludes post-conception primary infection and indicates a low risk of vertical transmission [55]. We observed that three out of four women with stillbirth had low avidity HCMV IgG, and among three women with abortions, two had low avidity HCMV IgG, indicating the potential of HCMV in contributing to poor pregnancy outcomes.

The limitations of this study were that (i) patients were selected based on non-specific acute febrile symptoms and not on standardized case definitions of the described infections; this may have influenced the positivity rate of the samples collected for the assays, and (ii) the study did not investigate heterophile negative infectious mononucleosis caused by the human cytomegalovirus.

In conclusion, women reporting to have signs and symptoms of viral infections during pregnancy are likely to have been infected with the viruses. This might be associated with adverse pregnancy outcomes, which calls for the need of including the screening of viral infections in the antenatal package in the low- and middle-income countries where the burden seems to be very high.

## Figures and Tables

**Table 1:** Distribution of sociodemographic characteristics of the enrolled pregnant women in Mwanza.

Characteristics	Frequency/Median	Percent/[IQR]
*Age (years)*	24	[IQR 21–30]
*Residence*		
Urban	55	32.2
Rural	116	67.8
*Occupation*		
Small-scale business	81	47.8
Unemployed	48	28.1
Self Employed	42	24.6
*Education*		
Primary	139	81.3
Secondary	32	18.7
*Marital status*		
Married	154	90.1
Not Married	17	10.0
*Gravidity*	3	[IQR 1–4]
*Gestation Age (weeks)*	25	[IQR 20–32]

**Table 2:** Clinical features of viral infections among pregnant women in Mwanza.

Characteristics	Frequency	%
*Fever*		
No	26	15.2
Yes	145	84.8
*Rash*		
No	142	83.0
Yes	29	17.0
*Itching*		
No	146	85.4
Yes	25	14.5
*Joint pain*		
No	22	12.9
Yes	149	87.1
* Flu-like symptoms		
No	63	36.8
Yes	108	63.2
*Headache*		
No	33	19.3
Yes	138	80.7
*Myalgia*		
No	13	7.6
Yes	158	92.4

* Flu-like symptoms: Running nose, sneezing, sore throat, headache.

**Table 3:** IgG avidity index among participants.

Variables	Avidity Index	Participants	%
Low	<40%	10	14.29
Intermediate	40–60	42	60.0
High	>60	18	25.71
		Total 70	100

**Table 4:** Factors associated with ZIKV/DENV among symptomatic pregnant women in Mwanza.

			Univariate		Multivariate	
*Patient Characteristics*	*Positive* *n (%)*	*Negative* *n (%)*	OR [95% CI]	*p*-Value	OR [95% CI]	*p*-Value
*Age (Year)*	29.5 [IQR 24–34.5]	24 [21–30]	1.00 [0.97–1.04]	0.887	1.16 [1.02–1.32]	0.024
*Residence*						
Urban	2 (3.6)	53 (96.4)	1		5.03 [1.04–24.29]	0.044
Rural	14 (12.0)	102 (87.9)	0.36 [0.80–16.6]	0.096		
*Occupation*						
Business	7 (8.6)	74 (8.6)	1			
Unemployed	6 (15.5)	42 (87.5)	0.81 [0.20–3.32]	0.773		
Self Employed	3 (7.1)	39 (92.86)	1.51 [0.48–4.79]	0.484		
*Education*						
Primary	13 (9.4)	126 (90.7)	1			
Secondary	3 (9.4)	29 (90.6)	0.10 [0.27–3.73]	0.997		
*Gravidity*	3.5 [IQR 2–5]	3 [IQR 1–4]	1.16 [0.90–1.49]	0.257	0.80 [0.52–1.24]	0.320
*Gestation Age*	28 [IQR 21–35]	25 [IQR 20–32]	1.00 [0.97–1.04]	0.887		
*Fever*						
No	3 (11.5)	23 (88.5)	1			
Yes	13 (9.0)	132 (91.0)	0.76 [0.20–2.86]	0.679		
*Rash*						
No	14 (9.9)	128 (90.1)	1			
Yes	2 (6.9)	27 (93.1)	0.76 [0.15–3.15]	0.679		
*Itching*						
No	14 (10.0)	132 (90.4)	1.000			
Yes	2 (8.0)	23 (92.0)	0.82 [0.17–3.89]	0.801		
*Joint pain*						
No	1 (4.6)	21 (95.5)	1		1	
Yes	15 (10.1)	134 (89.9)	2.35 [0.29–18.74]	0.420	2.46 [0.30–20.28]	0.840
*Flu-like symptoms*						
No	5 (8.0)	58 (92.1)	1			
Yes	11 (10.1)	97 (89.8)	1.32 [0.44–3.98]	0.627		
*Headache*						
No	4 (12.1)	29 (87.9)	1			
Yes	12 (8.7)	126 (91.3)	0.69 [0.21–2.30]	0.546		

## Data Availability

All data generated/analyzed during this study were included in this manuscript.
